# 1504. Weight Gain and Weight Loss Attempts among People with HIV(PWH) in Louisiana

**DOI:** 10.1093/ofid/ofad500.1339

**Published:** 2023-11-27

**Authors:** Lauren Richey, Jonathan Schroeder, Kyle Mistretta, Hui-Yi Lin, Connie Arnold, David Welsh, John Apolzan

**Affiliations:** LSU Health Sciences Center New Orleans, New Orleans, Louisiana; LSU / Our Lady of the Lake Early Intervention Clinic, Baton Rouge, Louisiana; Louisiana State University Health Sciences Center New Orleans, New Orleans, Louisiana; Louisiana State University Health Sciences Center, New Orleans, Louisiana; LSU Health Shreveport, Shreveport, Louisiana; Louisiana State University Health Sciences Center, New Orleans, Louisiana; Pennington Biomedical Research Center, Baton Rouge, Louisiana

## Abstract

**Background:**

Weight gain is common among PWH. The Louisiana Translational Collaborative on Health Behaviors [LATCH] is a multi-center workgroup created to study modifiable health behaviors in PWH in Louisiana with participating HIV clinics in three cities: New Orleans, Baton Rouge, and Shreveport. We set out to characterize the BMI, weight gain, and weight loss attempts within this population.

**Methods:**

PWH aged ≥ 18 y were recruited from three HIV clinics during regular appointments from 2019 to 2023. Participants completed standardized surveys that assessed weight gain/loss and methods used. Participants were divided into two groups: BMI < 30 and BMI 30 and greater for analysis.

**Results:**

106 PWH were enrolled as of Jan 2023. The average age was 51 y, most were African-American (81%), men (55%), and had a BMI >25 (74%). Fifty-eight (55%) participants had a BMI < 30, mean BMI and weight were 25 kg/m^2^ (range 17.9 to 29.8) and 75.6 kg, respectively. In the 48 people with obesity (45%), the mean was BMI and body weight was 38 kg/m^2^ (range 30 to 54) and 116 kg, respectively. Among the participants with a BMI < 30, 45% reported weight gain in the last 12 months, 7% reported trying to lose weight, but 31% lost weight, with a mean weight loss of 12.5 pounds. Exercise, medical illness, and diet were the most common contributing factors in the weight loss for this group. Among participants trying to lose weight, the average weight loss was 14 pounds. Among participants with a BMI of >30, 62% reported weight gain in the last 12 months, 27% reported trying to lose weight, but 40% lost weight, with an average of 23.7 pounds lost. Exercise and diet were the most common contributing factors in the weight loss for this group. Among participants trying to lose weight, the average weight loss was 26.7 pounds. No patients in either group reported weight loss surgery as a contributing factor in their weight loss.

**Conclusion:**

In this study, most people with HIV in Louisiana had a BMI >25 and reported weight gain in the past year. In the group with obesity, over a quarter reported trying to lose weight and those who tried had success. People with HIV and overweight or with obesity show encouraging outcomes when attempting weight loss, which may be critical in addressing the weight gain associated with first line HIV regimens.

**Disclosures:**

**John Apolzan, PhD**, WW(Weight Watchers) Inc: Grant/Research Support

